# Biological Agency: Its Subjective Foundations and a Large-Scale Taxonomy

**DOI:** 10.3389/fpsyg.2016.00041

**Published:** 2016-02-08

**Authors:** Adelina Brizio, Maurizio Tirassa

**Affiliations:** ^1^Faculty of Communication Science, Università della Svizzera ItalianaLugano, Switzerland; ^2^Department of Psychology and Centre for Cognitive Science, University of TurinTorino, Italy

**Keywords:** agency, interaction, phylogeny, cognition theoretical neuroscience, theoretical psychology

## Abstract

We will outline a theory of agency cast in theoretical psychology, viewed as a branch of a non-eliminativist biology. Our proposal will be based on an evolutionary view of the nature and functioning of the mind(s), reconsidered in a radically subjectivist, radically constructivist framework. We will argue that the activities of control systems should be studied in terms of interaction. Specifically, what an agent does belongs to the coupling of its internal dynamics with the dynamics of the external world. The internal dynamics, rooted in the species' phylogenetic history as well as in the individual's ontogenetic path, (a) determine which external dynamics are relevant to the organism, that is, they create the subjective ontology that the organism senses in the external world, and (b) determine what types of activities and actions the agent is able to conceive of and to adopt in the current situation. The external dynamics that the organism senses thus constitute its subjective environment. This notion of coupling is basically suitable for whichever organism one may want to consider. However, remarkable differences exist between the ways in which coupling may be realized, that is, between different natures and ways of functioning of control systems. We will describe agency at different phylogenetic levels: at the very least, it is necessary to discriminate between non-Intentional species, Intentional species, and a subtype of the latter called meta-Intentional. We will claim that agency can only be understood in a radically subjectivist perspective, which in turn is best grounded in a view of the mind as consciousness and experience. We will thus advance a radically constructivist view of agency and of several correlate notions (like meaning and ontology).

## Introduction

Most, if not all, research paradigms in psychology and in the cognitive sciences agree that the mind is a control system. If behaviorists acknowledged that minds exist at all, they would probably say that they are such systems. Classic information-processing and computational psychologists have often talked explicitly of the mind in such terms. Allen Newell, for example, one of the leading figures in classical cognitive science, wrote that the mind is “the control system that guides the behaving organism in its complex interactions with the dynamic real world” (Newell, 1990, p. 43). Artificial intelligence, Artificial life, and autonomous robotics have a necessary focus on control systems, and the same holds for the most recent trend in scientific psychology, namely the attempt at the integration and cross-fertilization of psychology and the neurosciences.

We basically agree that the main or exclusive function of the mind is to overview and control an organism's activities. However, the notion of *control system* is not easily defined, nor are the nature and the functioning of the particular control system that is the *mind*.

Newell's definition is cast in terms of *behaving organisms*. This definition is interesting under several respects. One is that it adopts a third-person perspective on the topic of action: generally speaking, no agent would conceive of its own activities and actions in terms of *behaviors*. Again, this is typical not only of classical cognitive science, but of most approaches to the study of control systems as well.

Second, Newell's definition entails that any control system that guides a “behaving organism” is a mind, that is that any animal endowed with a nervous system, from the Cnidaria or the Ctenophora (Hatschek, 1888; Moroz et al., 2014) to mammals has a mind, and that the main or only product of an organism's mind is behavior.

Our position diverges from Newell's on both issues. As regards the former, we will adopt a subjectivist perspective of agency, namely that agency can only be understood in the first person, and that the first person has causal powers, that is, it is not a mere epiphenomenon of non-subjective machinery endowed with “real” powers. However, the debate about subjectivity and its role is at least as old as psychology is, and we do not think that proponents of either position might be convinced to change their mind by a “silver bullet” argument of sorts. Thus, rather than arguing in favor of a re-evaluation of subjectivity, we will skip to the subsequent step and try to develop a possible description of cognitive architectures that may follow from such re-evaluation.

We will also object to Newell's implication that any control system from the simplest to the most complex is a mind. The problem with this notion is that it misses too many crucial features of what a mind is: in particular, the issues of meaning and Intentionality. We will propose that all agents are not equal, and that some differentiation be made between different types of biological control systems.

Still another crucial point of Newell's definition is that it talks of behaving *organisms*. It has often been claimed in classical cognitive science that minds may be embodied not only in biological bodies, but also in thermostats, computers, and generally in any physical and computational mechanism capable of supporting the basic operations of intelligence. This is a consequence of the *multiple realizability thesis* that follows from the computational postulate on the nature of the mind (see e.g., Turing, 1950; Haugeland, 1981; Pylyshyn, 1984).

While the ideas that we will discuss in this paper are incompatible with computationalism, we do not have the space here to engage in an examination and criticism of it (for which see e.g., Searle, 1980, 1992; Tirassa, 1994; Manera and Tirassa, 2010; Tirassa and Vallana, 2010). We will circumscribe our discussion to biological entities; it may be interesting, however, to notice that Newell (1990) himself, in apparent contrast with his own previous work (e.g., Newell and Simon, 1976), talks of behaving *organisms*.

Actually, one of the goals of this paper is to provide for a biologically based conception of interaction and agency, and to argue that this requires biology to take a step from its implicitly or explicitly eliminativist positions concerning the existence and the causal roles of subjectivity. Instead, we will claim that agency can only be understood in a radically subjectivist perspective, which in turn is best grounded in a view of the mind as consciousness and experience. We will also advance a radically constructivist view of agency and of several correlate notions (like *meaning* and *ontology*).

## World, environment, and interaction

Living organisms have several interesting properties that differentiate them from other entities. These can be resumed in the notion that living beings do not passively exist in the world, but actively interact with and within it.

The most apparent manifestation of this property is that they are capable of maintaining, at least within certain boundaries, their own coherence and autonomy in the face of a world which does not take particular care of them. This is not to say that the world is hostile toward them; sometimes it is, of course, but most of the time it is just indifferent.

*Coherence* means that living organisms have a substantially harmonious anatomic and functional structure, each part of which, under normal conditions, concurs to keep them alive and healthy and participates more or less congruously to the relations that they entertain with the surrounding world. *Autonomy* means that living organisms create and maintain an internal environment which follows dynamics of its own, that are neither completely separated from nor totally determined by the dynamics of the external environment.

Coherence and autonomy are not independent of each other; on the contrary, they shape each other in a dynamic circular relation which lies at the very foundation of life. Together, they provide for the adaptivity of organisms, that is, for their capability of creating and maintaining a dynamic compatibility with the environment in which they are immersed. When such capability falls under certain thresholds, the organism dies.

There are several ways in which living beings stay coherent and autonomous. One is by creating and maintaining a permeable separation between their internal environment and the external milieu. Indeed, it is because such separation exists that talk of an internal environment becomes possible at all. The internal environment differs from the external one in that it is structured (coherent) as well as in several physical and chemical features, which may range from temperature to the concentration of various substances like structural proteins, enzymes, nucleic acids, metabolic products and by-products, and so on. Permeability allows for an adaptive management of such differences, without the need for the organism to become a totally self-contained and self-sufficient universe, which would of course be impossible. In other words, organisms rely for their survival on precisely those world dynamics from which they have to keep, under other respects, a certain degree of separation.

Thus, the second way of maintaining coherence and autonomy is by exploiting the features of the environment that are relevant to the organism. Most living species, for example, are positively or negatively sensitive to degrees of light, temperature, or the concentration of various nutrients and toxins. Again, organisms have to rely on the external world in order to secure their autonomy, survival, and welfare.

A third way of maintaining coherence and autonomy is by exploiting the biological, behavioral, etc. features of other individuals, belonging to the same species or to others. This process may be viewed as just a special case of the previous one; however, given the importance that it gains in the species that adopt it, it deserves special consideration. Predation, symbiosis, or parasitism are obvious examples of how organisms may interact with each other. As interestingly, many organisms depend on each other for their own survival, as well as for the perpetuation of some parts of themselves, via sexual reproduction and related behaviors. Sexual reproduction in its turn imposes specific constraints on several features of the organisms, ranging from sexual dimorphism to each individual's need to be a desirable sexual mate. The possibility of relying on the others for one's own survival may become so prominent that the individual members of some species may lose their reproductive capabilities—as is the case, for example, with eusociality in social insects and other species where the biological unit is, under certain respects, the community—or end up sacrificing their own life so that other conspecifics may instead keep theirs.

We said above that living beings maintain their coherence and autonomy not thanks to isolation from the external world, but thanks to a delicate and dynamic interaction with it. They actually are immersed in a world with which they need to cope, if they are to survive and prosper.

The notion of *coping with the world*, however, is tricky. No organism could ever cope with all the features of the world. The world is too complex, too rich of dynamics that are more or less independent of each other, for any organism to keep track of the whole of it. It would anyway be extravagant to keep track of all the incidents and occurrences in the universe. Each organism can limit itself to cope with some of the external dynamics that are relevant to its internal environment, its survival and welfare, and possibly its interests and goals, at least as far as organisms with interests and goals are concerned.

This is not merely an issue of physical distance. It is trivially true that what happens in the Andromeda galaxy is practically irrelevant to the survival and welfare of a shrimp in the Atlantic ocean. The real point instead is that most of what “objectively” happens even in the shrimp's immediately proximal world is exactly as irrelevant to it.

The only happenings that do count for an organism are those that potentially affect its well-being, its survival, its reproduction, and its interests and goals. No organism could ever cope with all such occurrences, of course (if it did, it would never die); however, the more occurrences it can cope with, and the better it does so, the better its chances of prosperity are.

Each organism is thus capable of interacting with the occurrences and incidents of the world that are relevant to it; circularly, it is precisely *because* an organism can cope with such occurrences and incidents that they are relevant to it. What happens out of this set of world dynamics may affect the organism in several ways, at least from the viewpoint of an external observer capable of noticing the ongoing events, but will not be relevant from the organism's viewpoint. Think, for example, of radiations: since an organism does not interact with them, they simply do not exist to it, even if they kill it.

What is the nature of the occurrences and incidents that are relevant to an organism? Apart from a few physical and chemical parameters, most of them have nothing to do with what we consider the world's fundamental dynamics. We are accustomed to believe that the universe is made up of protons, neutrons, and electrons, or of even smaller particles, of electromagnetic waves and other forms of energy, and so on, and that world events are made up of movements, variations, and transformations of such entities and aggregates thereof. However, no control system of a living organism deals directly with electrons, atoms, or molecules (except, of course, for that of human researchers in fundamental physics, on working hours). What living beings deal with is air, food, preys and predators, rivers, trees and mountains, sexual mates, water, parasites, paths, obstacles, and dangers: all entities that, whatever their true or ultimate structure and composition, are nonetheless interesting as entities in themselves, characterized by dynamics of their own that are not reducible to those of fundamental physics and chemistry. And, of course, from the viewpoint of most organisms there just are no such things as *fundamental physics and chemistry*: as far as we know, these notions are characteristic of a comparatively small, historically given subset of the human species.

This means, in practice, that it is the organism itself that selects which, of all the happenings in the universe, are relevant to it: which peculiar configurations of atoms and energy are food, water, threats, opportunities, mates. In this sense, the environment is, to each organism, subjective: not because no external reality exists, independent of the organism, or because such reality cannot affect its welfare or its very existence, but because each organism is only capable of interacting with certain dynamics of the universe, with which it interacts according to the ways and ends that its nature allows.

Thus, a certain dynamics of the external world can be inconsequential to an organism, or influent but not relevant (because the organism is not equipped to interact with it—something that only an external observer might appreciate), or relevant in one way or another (being a threat, an opportunity, something edible, something with which to reproduce, and so on).

In other words, it is the internal dynamics of an organism that create the external ones and, in making so, give them meaning. Meaning is, therefore, subjective.

Each organism thus creates a certain set of external dynamical meanings with which it then interacts. The specific ways in which it does so are dictated by the biology of the species to which it belongs as well as, in part, by its own ontogenetic trajectory. It is the features of the organism's biology that establish its physical and chemical requirements, what kinds of geographical environment it will find more suitable, what food it needs and how it can recognize and obtain it, what dangers it has to defend from, whether, why and how it has to interact with its conspecifics, and so on. Within the same species, there always is room for individual variations. These may be due to small differences between the genotypes or between the phenotypes to which they give rise in their interaction with the environment (Lewontin, 1998), to learning, to individual preferences, and so on. However, of course, the similarities between the members of a certain species are much greater than the differences.

There can instead be remarkable differences between species. In evolutionary time, phylogeny has generated and selected many different ways to be in the world: all of them are equally subjective, all equally legitimate, all more or less equally compatible with the real world, all more or less equally capable of taking care of the organisms' explicit or implicit interests.

The nature of the relation occurring between an organism and its environment thus depends, in the last analysis, on the interaction between its genotype (which results from evolution), its phenotype (which emerges from the interaction between the genotype and the ontogenic environment), its idiosyncratic developmental paths, and the environment itself.

## The evolution of control systems

The range of processes that may fall under the label of “an organism's interaction with its subjective environment” is very broad. Multicellular organisms typically interact with the environment on many levels: from the exchange of water, ions, and metabolites, to processes aimed at recognizing and destructing non-self molecules and microorganisms, to the production of substances that may affect the metabolism, the growth or the behavior of a conspecific, an aggressor or a prey, and so on. While the final operative details of each of these functions are typically assigned to a specialized organ, apparatus, or system, all of an organism's parts participate, in one way or another, to the organism's interactions with the external environment.

In the phylogeny of animals, furthermore, an organ—or, better still, a whole system: the nervous system—has developed which specializes in the peculiar function of centralizing, governing, and coordinating, at least to a certain extent, certain features of the interaction with the environment.

The initial appearance and evolutionary success of this system were in the service of movement. To be capable of moving from a sunny area to a shadowy one and vice versa, or of actively searching for water and food or fleeing from a danger is extremely useful to an organism. This requires a behavioral coordination that can only be achieved by a specialized management of the relevant processes.

Circularly, the nervous system originated from sensory-motor circuitry for phototaxis and chemotaxis. Cells endowed with electric properties enabling them to react to light or chemicals progressively differentiated into what will become a system of specialized sensors and effectors and, later on, a system in charge of coordinating and mediating the management of the various contingencies and opportunities that the animal meets in the environment.

This type of circular relation is typical of biology (Mayr, 1997; Gould, 2002). Organisms do not evolve because the world forces them to do so: that is, they do not progressively become more adapt to an objectively given environment which “poses problems” that they must “face” by evolving (see also Gould and Lewontin, 1979).

Something very different happens instead: each organism creates, through its peculiar ways of interacting, a subjective environment of its own (an *Umwelt*, in Uexküll, 1934, words), and it is to this that it, circularly, is adapted. The “problems” that living beings must “face” are not in the world, but in their interaction with the world; analogously, the “solutions” that evolve are not in the organisms, but in the interaction that they have with their subjective environment. Thus, there are neither real “problems” nor real “solutions”: only more or less successful ways of creating and maintaining sustainable *Umwelten*. A significant genetic mutation can, under certain conditions, give rise to a new species, characterized by different ways of interacting with a different subjective environment.

Of course, each organism's subjective environment has to be compatible with the real world, whatever its ultimate nature may be. This is, indeed, a matter of compatibility, not of problem solving. Evolution is not a movement on the gradient of adaptation toward optimality.

Thus, as far as nervous systems are concerned, their appearance in phylogeny is by no means necessary: the “real world” does not “pose problems” requiring coordination and control to an organism which, having no such capabilities, finds itself in the need to evolve them. Actually, countless living species have no control system whatsoever, least of all one made of neurons. On the other hand, nervous systems, once appeared, significantly alter the types of interaction that organisms can generate. Coordination and control become salient features of such interaction, which gives rise to still different types of interaction, and so on. As a result, a whole evolutionary lineage takes a wholly new course.

By the same line of reasoning, not all control systems need be similar both in structure (which is obvious) and in the type of interaction they create and maintain with the world. We will propose an outline of two very large-scale types of such systems, which we will call *non-Intentional* and *Intentional*, based on an analysis of the possible types of interactions with the world. A particularly interesting subtype of Intentional architectures called *meta-Intentional* will also be described.

Intentionality, or aboutness, is a mind's property of being able to entertain semantic (meaningful) relationships with the world. In philosophy (e.g., Searle, 1983), artificial intelligence (e.g., Rao and Georgeff, 1992), ethology (e.g., Prato Previde et al., 1992), and cognitive science (e.g., Airenti et al., 1993; Tirassa, 1999b), Intentionality^1^ often is characterized in terms of mental states like various types of desires, beliefs, and intentions, with propositional content. This is often couched within the computational postulate about the nature of the mind, whereby cognition consists in the syntactic manipulation of symbols (Manera and Tirassa, 2010; Tirassa and Vallana, 2010).

The computational postulate, however, is far from being unanimously accepted, and other areas of literature tend to equate Intentionality with consciousness or phenomenal experience or, as it is often said, to view representations as happening at the interaction of the conscious mind/brain and the external world (see e.g., Heidegger, 1927; Merleau-Ponty, 1945; Nagel, 1986; Varela et al., 1991; Searle, 1992; Varela, 1996). This means, among the rest, that the mere neural coding of sensory stimuli does not count as representation (e.g., Clark, 2001).

This is also our view. For the analysis that follows, a simple definition of Intentionality as synonym with aboutness, semantics, and phenomenal experience will suffice.

## Types of control systems

What nervous systems do is to mediate in wholly new ways between the animal's internal dynamics and the external ones. Simple mobile animals are characterized by taxes, that is, movements in space along physical-chemical gradients such as light, temperature or the concentration of certain molecules. When nervous systems appear in phylogeny, taxes are substituted for by locomotion, that is, active movements, endogenously generated from internal states like the variation of certain physiological parameters, the perception of relevant entities in the external world as well as, a few millions of years later, desires and opinions.

An animal's internal dynamics end up being the very center of its interaction with its subjective environment. This leads to the differentiation of several types of internal dynamics, and therefore to a progressive increase in the structural complexity of the interaction that the animal is capable of generating.

Of course, the control systems of animals are not all alike. The very anatomy and physiology of control systems are strikingly different across species. Differences in the types of interaction generated follow accordingly.

### Non-intentional control systems

The evolution of nervous systems may be described in several ways. In principle, each such description, if correct, should match the others; however, current knowledge is far from providing maps of such precision. What is available is, on the one side, a great deal of punctiform analyses of single behavioral or cognitive functions in single species and, on the other side, a few rough decompositions as tentative identifications of wide phases in the phylogeny of control systems. Our discussion will fall into the latter area: based in part on previous work (Tirassa et al., 2000) we will propose an extremely large-scale classification of control systems, from the viewpoint of their (hypothetical) subjective functioning.

After the appearance of control systems, the next crucial step corresponds to the transition from invertebrates to vertebrates, characterized among the rest by the appearance of encephalic and cortical structures (Gans and Northcutt, 1983). Such anatomic transition is likely to find a functional correspondence in the appearance of phenomenal experience in the proper sense, that is, of consciousness, or awareness, or at least of object-based cognition, which is its primary manifestation. Despite several studies (e.g., Menzel and Giurfa, 2001; Menzel et al., 2006; Bateson et al., 2011; Mendl et al., 2011; Gibson et al., 2015), we still do not know whether and how invertebrates are conscious. Even if they were, however, there would probably be nothing that they would be conscious of; at least, nothing in their behavior lets us think so (see e.g., the discussion of phonotaxis in crickets in Clark, 2001; Hedwig and Poulet, 2005; Hedwig, 2006; Hennig, 2009). Even in the apparently most complicated cases (such as the famous “dance” of honeybees) their behavior can invariably be explained in terms of comparatively simple transformations of sensory signals into motor commands (see e.g., Kesner and Olton, 1990; Wehner, 2003; Poulet and Hedwig, 2005).

Winged insects, for example, prepare for landing as a reaction to the visual expansion of a texture from below, signaling a surface rapidly getting closer. Analogously, they prepare for flight as a reaction to the contraction of a texture from below (signaling a surface rapidly getting farther) as well as to the expansion of a texture from above (signaling a potential danger approaching; Lindemann and Egelhaaf, 2012). This non-object-based mechanism is comparatively simple and extraordinarily effective; so simple and effective, indeed, that it has not undergone significant evolution over the last several million years.

However, nothing in the behavior of winged insects makes us think that they have any semantics for, or conscious experience of, surfaces for landing or of dangers approaching. To them, a texture is worth another, provided it activates the takeoff or landing mechanisms. They appear to be completely unable to discriminate between a rose petal falling and the newspaper with which an exasperated human is striving to kill them.

If these animals are conscious, we are unable to understand *what* they are conscious of, and, therefore, *why* they should be conscious at all. One hypothesis that could be made is that consciousness might be a necessary property of any neuronal system (but then, even of a neuron in isolation?). Such hypothesis, however, is not necessarily better than others, nor would it explain what such animals could be conscious of.

### Intentional control systems

Intentionality is the property of entertaining semantic, that is meaningful, relations with the world (Brentano, 1874). Its appearance is a big turn in phylogeny: while the case of invertebrates is uncertain, it can safely be claimed that vertebrates are conscious because they experience the world, that is they give meaning to it. Intentionality can only be conscious (Searle, 1992): experience requires a point of view (in a sense, experience is a point of view) and a point of view has to be *someone's* point of view, therefore subjective (Nagel, 1986). Thus, there is no experience without subjectivity and, of course, no subjectivity without consciousness.

This conception is different to the general consensus in mainstream psychology. Most scientific paradigms from the nineteenth century to the 21st build on the assumption that consciousness is substantially irrelevant, or only marginally or occasionally relevant, and that what really matters is unconscious, non-subjective, non-meaningful knowledge and processes. Although a thorough discussion of these issues would fall outside the scope of this work, it may be useful to spend a few words.

In the perspective we are trying to outline, the mind is neither an epiphenomenon (as it is in behaviorism, neural reductionism, and related forms of eliminativism) nor a set of descriptions (as it is in classical cognitive science and computational psychology). Both such accounts are untenable for philosophical (e.g., Searle, 1980, 1992; Nagel, 1986; Johnson, 1987; Varela et al., 1991) and biological (e.g., Edelman, 1992; Varela, 1996) reasons; furthermore, they are, in a sense, equivalent, in that both are rooted in, and possible consequences of, dualism (Tirassa, 1999a). Instead, the mind is a material property of the brain, which means that cognitive causation does not go from brain to brain and from brain to mind, but from mind/brain to mind/brain; better still, since brains do not grow in vases, from mind/body to mind/body.

That we have no hint at how this is possible, that is, at how a few kilograms of seemingly undistinguished matter may have the property of being subjective, does not make it less real. Actually, all existing theories are as obscure on this point: how does subjectivity occur as an epiphenomenon? how does it emerge from computation? Therefore, instead of endlessly arguing in favor or against the various views, we will just try to go on, trying instead to develop a few consequences of our approach, from which it might be judged more aptly.

To equate mind, consciousness, subjectivity, meaning, and experience also means that there is no Self, if the word is taken to refer to a *homunculus* or mental entity which is abstracted from, and exists independently of, space and time. Instead, the mind re-creates itself from instant to instant. The sense of continuity that we perceive depends on the fact that each “slice” of such re-creation is causally generated by the meshing of the preceding one and the current interaction with the world, and so on, back in time, up to the very first instant when our mind began to exist.

Each such “slice” in the functioning of a mind/body literally is the product of the previous history of that mind/body, plus its current interactional dynamics. The patterns with which the mind/body re-creates itself are rooted in the evolutionary history of the species to which the organism belongs. At least in certain species, such patterns also depend on and, circularly, generate individual differences whose roots are to be found in genetic variations between individuals as well as in the details of each individual's interaction with the world (that is, its ontogenetic history). In a metacognitive species like ours, the latter includes the mind's interaction with itself (that is, its autobiography) and with other minds and the artifacts they generate (that is, social and cultural history).

Another point worth remarking is that this conception of the mind requires that it be identified neither with attention (at least because we are conscious of several things on which we do not focus our attention) nor with abstract or formal reasoning, language, or self-awareness. The latter capabilities depend on the existence of consciousness but are not identifiable with it, both for analytical reasons and because most Intentional species do not appear to possess them.

## Interaction and behavior

Nervous systems, we said, are control systems. What they control is not, as in Newell's definition cited in the introduction, the organism's *behavior*, but its *interaction*; or, at least, certain features of the overall interaction. There are many reasons why this remark is crucial.

The first is that behavior is a third-person term. Behavior only exists in the eye of an observer who pursues interests of its own, not in that of the “behaving” organism. Control systems do not behave: they work in the first person.

Actually, non-Intentional control systems only work in the first person in a very peculiar sense, because, as we saw, there is no reason to think that “there is anybody home.” Notwithstanding, what such systems produce is interaction anyway, but one of a kind that consists in the mere activation of motor patterns starting from the meshing of sensory patterns with relevant physiological internal states.

Another reason why control systems are better said to produce interaction than behavior is that the nervous system does several things beside “producing behavior” (whatever acceptation the term is given) or even “reasoning.” Many bodily functions fall, wholly or partly, under its jurisdiction: it controls, for example, the activity of the cardiovascular, the respiratory, the digestive, and the endocrine systems.

These activities are not independent of, or separate from, the generation of interaction. Notwithstanding their anatomical, physiological, and functional variety, the components of the nervous system work in strict synergy, which, of course, is precisely why it is a *system*. All of them are directly or indirectly interconnected, so that the nervous system is best described as a single interaction-producing network of cells. While some parts of it are more involved than others in the various aspects of interaction, it certainly is not an assemblage of “encapsulated modules” working in isolation from one another.

Consider, for example, what happens to a mammal who perceives a predator—say, a gazelle and a lion. For a start, such “perception” is mental activity: what we mean when we say that the gazelle perceives the lion is that it views a certain entity in the world as a specific type of potential threat to its security. To say that this experience is subjective does not imply that the lion literally is a creation of the gazelle's mind, but that its meaning is. “Predator” is a semantic relation between the two animals in the current situation, not an intrinsic property of one of them: to an elephant, a cat is much less a predator than it is to a mouse; to the mouse, a lion is much less a predator than it is for a gazelle; to the gazelle, a lion on the horizon is much less a predator than it is a lion 100 m away. To a hypothetical gazelle equipped with an armor plate and pump-action rifle, the lion would be nothing more than an occasional nuisance^2^.

When an animal perceives such a danger, its blood pressure rises, as the result of a variation in heart activity and in the total caliber of its blood vessels. Furthermore, there is a redistribution of the blood flow away from certain districts (like the digestive system) and toward others (like the brain and the locomotor system). Respiratory frequency increases. Several hormones and other substances are released in the blood and go to affect the functioning of an array of organs and apparatuses. As a result of these complex modifications in its mind/body, the animal will become prepared to a fight-or-flight activity. Such condition will modify in turn the subsequent flow of the animal's mental dynamics; e.g., it will be frightened, but also more ready, compared to what would have been in a different situation, to search its subjective environment for certain relevant affordances. The gazelle might, for example, act so to draw the lion's attention away from its offspring; it might recognize a river not as a reservoir of drinkable water, but as an obstacle for its enemy, who might be reluctant to cross it; it might see the herd as a source of salvation and safety, and so on.

Talk of instinct here would be correct and misleading at the same time: correct, because the reconceptualizations that the gazelle does of its subjective environment can hardly be viewed as the result of sophisticated reasoning, or of preceding experience with similar situations (although, of course, the latter may certainly play a role). Misleading, because it is extremely unlikely that the control system of the gazelle is hardwired to do things like “looking for salvation beyond the river if a lion is behind me, the herd is too far removed, and the river is wide enough to be a problem for it; but only if the offspring is safe.” This would only be possible in a giant lookup table like those of computational psychology, and mind/bodies simply are no such tables (if only because there is no *homunculus* inside who could look them up). Without representations, no component of the gazelle's subjective situation (the lion, the herd, the river, and so on) could be present to its control system; and, without a however small leap of intuition and creativity, no acknowledgment of the possible moves and their comparative chances of success, and therefore no situated decision, would be possible.

Thus, the notion of instinct is of little help. What happens is instead that the animal continuously reconceptualizes the surrounding environment. This is certainly made possible—indeed, generated—by the gazelle's specific biology, comprised of its phylogeny and ontogeny, but no less mental for this. What is misleading about the notion of instinct is the impossibility for such label to capture the actual nature of Intentional control systems. The advantage that such systems offer, compared to non-Intentional ones, is precisely that they work on dynamic flows of meanings, not on hardwired sensory/motor relations.

Reconceptualization means that a tight semantic coupling to the world is maintained. Meanings are not in the world, but in the animal's experience in each moment. The animal's past history is crucial in the generation of its current experience, not because it has been coded and stored for future reuse, but because it is what has led the animal to the particular state in which it currently is. The mind exists exclusively in the present, but such present is the child of the past, and results from the integration of the past with the world as it is now (Glenberg, 1997).

An agent's cognitive dynamics across time results from the interaction of its mind/body with the surrounding (mental, bodily, physical, and social) environment. Interaction at any instant t_*i*_ is causally generated by the state in which the mind/body was in the instant t_*i*−1_ that immediately preceded, together with co-occurring factors that may affect its functioning, like the activity of sensory receptors, emotional and thinking processes (whatever they are in each species), the effect of various blood chemicals, and so on.

Each “slice” of a mind/body dynamics thus is the product of that dynamics so far, meshed with the meanings found in the interaction in which the animal is currently immersed. This way, it is neither a mindless body nor a disembodied mind that causes the overall dynamics: the state of an agent's mind/body at any slice of time plays a causal role in the state of that mind/body at the subsequent slice of time.

In its turn, interaction at t_*i*_ will contribute to generate the state of the mind/body in the instant t_*i*+1_ that will immediately follow. Thus, in each instant interaction results from all the interactions at t_*i*−*n*_. Memory and learning are to be understood as modifications of each possible future experience, rather than independent, switch-on/switch-off “cognitive functions.” The pattern of development of such history results from the biology of that particular organism, so that it definitely is not a matter of nature vs. nurture (or of rationalism vs. empiricism) that we have here. Viewed from a biological vantage point, these are false dichotomies (see, for example, Lorenz, 1965).

Intentional systems thus are in no way “less biological” than non-Intentional ones, unless one believes that biology needs to be eliminativist, and such eliminativist biology is then opposed to a mentalist psychology supposed to have nothing to do with biology. That this conception has ruled both disciplines for several decades is the consequence of the acceptance, on both sides, of Cartesian dualism and its legacy. There is no reason to accept such position; indeed, there are several reasons to reject it.

Let us now go back to Serengeti. The gazelle that is fleeing from the lion is not “behaving”: it is giving an overall meaning to the subjective environment in which it finds itself, and reconceptualizing in its light the environment itself, looking for affordances relevant to such meaning.

Of course, the lion who is chasing the gazelle does, in its own way, the very same.

## Agents

We can now define an agent as an Intentional, conscious organism who lives in a situation, and strives continuously to make it more to her liking^3^. What we call the *situation* is a subjective, dynamic, and open map of the world.

This definition allows to exclude several entities that, albeit self-propelled, do not act in any sense of the term (if not, possibly, in a metaphorical one): household appliances like the thermostat that operates the air conditioning in this room, the computer on which we are writing this paper, or, in a very different fashion, the mosquito that, aware of nothing, is flying around us, trailing the heat of our body and the carbon dioxide that it produces. Yet, each of these entities or living beings has been characterized as an agent proper in other paradigms within the cognitive sciences.

In our definition, an agent can only exist in biology if it entertains with the world the kind of relation that Maturana and Varela (1980) call *structural coupling*. The Intentional features of this relation were discussed in a previous section, as well as the remark that they must satisfy a constraint of compatibility, not correctness, with respect to the real world.

One implication of the latter consideration is that several different types of agents can exist in principle, and do indeed exist on this planet. Actually, there exist as many types of agents as representational species, and smaller differences occur between the various individuals that belong to each. Each (type of) agent will see its own set of world dynamics and possibilities for action.

Human beings assume that they can entertain objective knowledge because they are capable of generating descriptions of the world and exploiting them for action as well as for intersubjective, publicly shared, and agreed-upon consideration. However, this is only our specific way of knowing, compatible with the ultimate truth (Kant's *Noumenon*, 1781) but neither closer to it nor more objective than that of other species (Nagel, 1974, 1986).

Let us consider again the coupling between an agent and its world. What is coupled is the agent's internal dynamic and the external ones. The world has dynamics of its own, which depend on its properties at the various levels that can be considered: chemical, physical, geological, meteorological, astronomical, biological, and so on. For the scope of this paper, however, it will not be necessary to discriminate between these diverse entities: we will just gather them all under the label “external dynamics.”

An animal's internal dynamics include the meanings that it finds in the external ones; circularly, the external dynamics may be said to be generated by the internal ones. Each external dynamics thus corresponds to one of the entities that are interesting for the animal and, while such entity is present to the animal's mind (that is, while it subjectively exists), it is a continuous flow of mutable, self-modifying meanings.

It is necessary to conceive of such dynamics as *flows* because the world is mutable. To the gazelle, a small dot which is rapidly getting closer can suddenly turn into a lion, but then it may begin to chase another member of the herd, or it may reach too close to the place where the younglings are. The river may be too rapid to cross, but then open into a slower bend that permits safe wading; but, with the lion getting closer, the perceived dangerousness of the rapid trait may decrease to the point that attempting to cross it becomes preferable to being killed. A control system has to view the world as flows because the world is a flow, and even more so is the subjective environment in which an agent lives.

It is necessary to conceive of such dynamics as flows of *meaning* because what counts is the meaning of the various entities, that is, the role that they play in the agent's overall situation and the actions they afford. Control systems are not there to dispassionately, disinterestedly compile inventories of the entities that exist in the universe, but to do something with them: eat them, fight them, take care of them, ignore them, have sex with them, and also—why not, for a species like ours?—put them into inventories, but meaningful ones. The situated roles and affordances that characterize each entity are not separate from the entity itself, or a later attachment to an otherwise objective, neutral knowledge: instead, they are the very reason why the mind exists.

It is necessary to conceive of such dynamics as flows of meaning *within an overall situation* because each flow exists not in isolation, but relative to the others that the animal “views” in each moment. The meanings that each flow has in each moment depend on the current state of the overall situation and in their turn contribute to establishing it. The gazelle who is running from the lion will view the river as an opportunity for salvation, rather than as a good place for a rest and a drink, not because its mouth is not dry, or because the water in the river ceases to be drinkable when there are lions in the neighborhood, but because the point of the situation has nothing to do with mouth dryness and drinking. Thus, it is the dynamic meaning of the overall situation that gives the river its meaning as possible salvation, and running toward the river modifies the overall situation. It may, for example, give a sense of imminent salvation that has the gazelle double its efforts, while finding itself in the wide, open land might give it a sense that salvation is out of reach, and thus induce it to accept an opportunity for fighting instead.

## Actions

An agent lives in a complex situation, made up of dynamic flows of meanings.

Each such flow may subjectively be more or less pleasurable; or it may be neutral, which only means that it is neither particularly pleasurable nor particularly unpleasant. Most of the times, a flow will be more pleasurable under certain respects and less under others. The greater or lesser pleasantness of each dynamics depends both on the dynamics itself and on how it fits into the overall situation^4^.

To act is to alter such flows so to make the overall situation more pleasurable. The change in the animal's overall situation depends on specific interventions upon specific features of the subjective environment. Each flow of meaning that contributes to making up the overall situation may offer opportunities for action; to intervene upon one or another such flow depends on their respective pleasantness, on their respective contributions to the pleasantness of the overall situation, on the apparent possibilities for successful action and, all in all, on the balance of contingencies and opportunities that the agent views in the world.

Since the world is dynamic, and follows causal paths of its own, to act is to interfere with one or more such paths so to alter its spontaneous evolution. An action thus is an induced modification of the dynamics that the subjective environment would otherwise undergo. Since the agent's capabilities for action are limited, the agent will focus on one such dynamics, or on a few, leaving the others to their natural course.

Furthermore, since the world is dynamic, to act requires monitoring its spontaneous evolution, interweaving one's own moves with it and managing to dynamically coordinate the relation between action and world. This requires at least a minimal capability of prediction of what the evolution of the world will be with or without the agent's interference, or with different possible interferences. Action is intrinsically situated: were it not so, there would simply be no action at all. Of course, these capabilities of monitoring, prediction, and coordination will be different between species (and, within each of them, between individuals). Each species lives in its own type of subjective environment, and acts within it.

Since the subjective situation is mutable, the agent will move from one flow of meaning to another, always trying to make the overall situation more pleasurable. This process is continuous and seamless. Exactly as the world is a continuous dynamic flow, so are the agent's mind and actions.

Actions, thus, have no beginning and no end other than the points in time when the agent sets their beginning and their end; and they are not chosen out of a repertoire which univocally defines their preconditions, effects warranted by default, and procedures of execution, as it happens instead in most classic theories of planning, both in psychology (e.g., Newell and Simon, 1972; Shallice, 1982) and in classic artificial intelligence (e.g., Fikes and Nilsson, 1971; Russell and Norvig, 2009), or even in ethology, with the notion of ethogram (Jennings, 1906; Makkink, 1936). The surfer who rides the ocean waves, exploiting their push, trying to keep her balance by simultaneously *following* the waves and *fighting* them, provides a better metaphor of an agent's life than the game of chess does, with its discrete and precisely defined moves carefully picked out of a closed repertoire and staged in a closed world, where nothing happens except the moves themselves, one at a time.

In other words, there is no intrinsic ontology of actions, except for the one that the agent will throw in at each moment. Similarities between situation/action couples, of course, allow an observer to generalize and abstract, but that does not mean that such generalizations capture a natural subjective ontology. The subjective ontology of action will depend, moment by moment, on the situation in which the agent finds itself and on the interests that it pursues in it.

This shows particularly well if we consider what might be called *the granularity of actions*, or, better still, *the minimal unit of action*. When we say that “an agent is doing something,” what do we mean, precisely?

What the agent does is to alter, in a direction which it foresees as favorable, the spontaneous evolution of the world, by leveraging on one of its characteristics. The ontology of the representation that the agent has of the world dynamics are not predefined, but they are created, moment by moment, according to the agent's interests and to the contingencies and opportunities that it views in the world. The same holds for actions, which are the external counterparts of representations. The ontology of action is created moment by moment, because that is also how what the agent represents is generated.

This may be viewed as a reformulation of the idea that what a representational animal does is to live within its situation, not to behave in greater or lesser accordance to the descriptions that an external observer might give. Furthermore, there can exist no repertoire of possible actions stored in an unconscious subsystem placed out of the here and now, if only because no stored recipe could be coupled to the current state of the world.

Thus, there also is no fixed minimal unit of action; at each time, the minimal unit of action will be what the agent decides it to be. To look inattentively at a landscape from which no danger is expected, while slowly grazing in the grass, is as much an “elementary” and “unitary” action as it is to focus in sudden alarm on a particular dot in the landscape, wondering whether it could be a lion.

Nor would it be a good idea to consider “elementary action” the minimal body movement possible to an individual. This would make no sense from the psychological or the physiological points of view, because we do not usually reason in terms of physical movements (except when we are learning a new movement, or when a breakdown occurs during action) and because to define such minimal movement would be impossible: the activation of a single motor neuron? and on what temporal scale?

The perspective that we are trying to outline relies on a radically non-dualist conception of the mind/body. The subjective situation in which the agent finds itself includes at least (for several species, *only*) visual, auditory, olfactory and other types of perceptions, as well as proprioceptive information concerning posture, what parts of the body are in touch with what, and so on. Such information may have variable degrees of granularity, according to the global properties of the situation and to the contingencies and opportunities that the agent views within it.

To act is to coordinate these information with the decision one is making, reconceptualizing at each moment the position that the head, the eyes, the limbs and the rest of the body should have. The realization of an action is thus the direct counterpart of the intention to perform it: it requires nothing more than that, nothing that is not already “contained” in the intention.

This makes no sense in a dualist “mind sends commands to body” perspective or in an eliminativist “mindless body moves according to instincts, neural firing, or reinforcements” one. However, it becomes reasonable in an Intentional view of the mind/body as one of the material properties of a control system which includes the whole nervous system, as well as its relations with the rest of the body and the surrounding environment^5^.

Thus, what happens is not that the agent represents a goal and, while time magically stands still, searches an inner store for the action(s) that will provably realize such goal, and sends the decision through a descending hierarchy of “levels of abstraction” until it somehow is translated from the cognitive into the bodily, becoming a sequence of commands delivered to the effectors for execution. What happens is instead that the agent singles out, in its subjective environment, a certain dynamics which offers some desirable opportunity (what might be called an attractor), and in so doing it reconceptualizes the whole of its own mind/body system in the realization of the relevant intervention, remaining at each moment coupled to the dynamic subjective environment. Perception, decision, action, and feedback are not different phases, possibly assigned to different subsystems, but different viewpoints that an observer may take of the mind/body, while the mind/body simply coordinates with the world.

This conception owes a lot to Gibson's (1977, 1979) notion of *affordance*. In a possible reading of his work, the entities of the world present themselves as attractors, variously positive or negative, that are afforded (hence the neologism) to the animal. Affordances are neither in the world nor in the animal: they reside in the interaction between the two. What the world puts in the interaction (and makes Gibson talk of *direct perception*) is the resources and the constraints to which the animal's control systems must conform, such as the invariants of the optical flow. What the animal puts in the interaction is its own nature, which makes a certain configuration of light, a certain texture, and so on, take the subjective shape of a certain affordance^6^.

## Meta-intentional architectures

We defined an Intentional agent as a conscious organism who lives in a subjective, open, and continuingly revised interpretation of an ultimately unknowable environment—what we call the agent's *situation*—and strives to make it more to its liking. The agent's mind is the experience of a complex flow of meanings, and meanings are dynamical affordances.

Let us now go back to the control systems of animals. In our extremely large-scale theory of the phylogeny of control systems, the first big transition which they underwent is the appearance of representations. The second is the appearance, in one or few evolutionary lineages within mammals, of what we will call *meta-Intentional* control systems.

Let us start from a related notion, that of *metacognition*. This term was first introduced by Flavell (1979), who defined it as the ability to think about thinking, and has since been used mostly in the area of human social cognition and communication (e.g., Tirassa and Bosco, 2008). However, it is misleading insofar as it seems to refer to a set of capabilities that make up a supplementary, “upper cognitive layer” that adds to a “base cognitive layer” without actually changing the meaning of the latter, but simply manipulating it or exploiting it when needed. This is the case, for example, with logical and formal metalanguages, upon which the notion of metacognition is framed. Such conceptions, however, cannot be applied to psychology or biology, precisely because they rely on a propositional (that is, formal, syntactic, and recursive) notion of mind, which could only work under the assumption that there is a *homunculus* inside who is in charge of operating the system, knowing when and how to nest the propositions, how to manipulate them, and so on, meanwhile losing meaning (Searle, 1980).

The mind is one; it is not composed of layers, least of all layers of nested computations. “Metacognition” can in no way be independent of, separate from, or placed above an alleged “rest of cognition”; on the contrary, it is intrinsic to the human way of knowing the world, that is, to the internal dynamics of the human mind. The dynamics that we see in the world, their pleasantness, and the affordances that they offer are immediately and intrinsically made different by such capabilities.

In our proposal, meta-Intentionality refers to a whole constellation of interwoven capabilities that can be resumed as the idea that the individual itself—including its body, its mind, its history, and so on—becomes part of what is represented. Roughly, while Intentional control systems can be said to experience the world, meta-Intentional ones can be said to experience themselves in the world.

Meta-Intentional minds actually are a subset of the Intentional minds, but one that is enough interesting to warrant a separate discussion. Because our comprehension of the minds of the other primates and of the cetaceans still is so unsatisfactory, the only known species that can safely be said to belong to this class is ours; however, that at least one meta-Intentional species exists is enough to require consideration.

The mind of a meta-Intentional agent has as its object of experience the agent itself, immersed in and interacting with its subjective environment as it is, was, or could be. This flow is based upon a narrative infrastructure which includes “islands” of description and explanation of the meaning themselves. Such descriptions and explanations offer further opportunities for actions, or affordances.

Suppose you are arriving at a friend's party. As you enter, the host introduces a person to you; she smiles and pronounces her own name; you pronounce yours and shake hands with her. After a brief exchange, you go on to meet the other guests. Some of them you know, some you don't, so you probably get introduced to a few other persons. After a while you chance upon the person whom you met on your arrival. Under normal circumstances, you recognize her face and start the conversation from where it had been interrupted, maybe searching your memory for her name. The next day you meet your friend for lunch, and she is again in the company of that person. If memory does not fail you, this time you effortlessly remember her name and begin a friendly conversation, reminiscing about the main events of the party and letting the exchange go wherever the three of you let it go. If you and she become acquainted, you will end up recognizing her at a distance, from her way of walking and the general shape of her figure.

What has happened? When you first met this person, at the common friend's place, you paid attention to (at least) basically two things: her look—particularly, her face—and her name. Like all human beings, you have a specific faculty of face perception and general person recognition (Paller et al., 2003; Peterson and Rhodes, 2003), which will of course take due notice of that person's lineaments and name. In more detail, when she is introduced to you you *experience* her look and her name; this experience becomes a *description* of that person, which in its turn *shapes your experience* of that person the next time(s) you meet her, allowing you to recognize her with increasing certainty. What we have here is a circular (better still, spiral) relation between “base-level” experience and “upper-level” metacognition.

Meta-Intentionality is not necessarily reasoning: it is just the intertwining and coevolution of experience and description that allows for the re-enactment of the cognitive performance (Guidano, 1987, 1991). Experience gives rise to descriptions in the form of narratives, explanations, maps, and so on; these become reincorporated into experience giving it new forms, structures and meanings.

Of course, this process may occasionally become more deliberate and ratiomorph, e.g., if the second time you meet that person at the party you have forgotten her name, you might actively conjure up a way to have her say her name again; or, if you find yourself interested in her, you might actively try to build an understanding of her ways of looking at the world, her interests, and so on. The point is not that these activities are not possible, but that they are not necessary. In time, the very infrastructure of your experience of that person will be shaped by the maps of her that you have built, so that under many, or most, circumstances, you will know, with no particular attentional or reasoning efforts, how you ought to behave with her, how she would react to something that you might say or do, what you can expect from her, and so on. Your descriptions will have melted into your experience, changing its shape and allowing for new, more complex maps to emerge.

Something very similar happens when we substitute the notion of explanation for that of description we have just used. A meta-Intentional mind, or at least that which characterizes the human species, is structured so to look for explanations of the events it perceives. Such explanations may be couched e.g., in folk psychology (e.g., Tirassa, 1999b; Tirassa and Bosco, 2008; Bosco et al., 2014; Brizio et al., 2015), naïve physics (e.g., Hayes, 1979; Smith and Casati, 1994; Spelke, 1994; Smith, 1995), and related ways of looking at the world. Here, again we have the same spiral relation between experience and explanation; of course, it would be reasonable to argue that explanation is indeed but a type of description.

Thus, when we sit in a car and turn the key we have a whole, complex set of expectations and experience. If the engine does not start our mind begins to formulate dynamics of possible explanations. This happens because we have had different kinds of experience with engines that start and do not start. Such experience, which begins partial and scattered, progressively becomes more unitary and, interwoven with fragments of descriptions and explanation, goes to shape the future experience, which will present itself already laden with partially ready-made cognitive structures that in their turn allow a more sophisticated set of possible action. When the engine does not start, an expert driver will immediately look at the dashboard to check if it is turned on, she will try to remember when it was that she had the battery checked last time, if there is petrol in the tank, and so on. Each such affordance is made possible by the narrative structuring of experience which is provided by description and explanation.

Another crucial feature of human meta-Intentional agency is plan construction and use. A plan is a resource for action (Agre and Chapman, 1990), a description that we use to guide our management of the situation. To build a plan is to imagine an alternate situation, or several alternate situations, and to keep it present to our attention while we look for a way to realize it.

At first sight, plans are the meta-Intentional version of what simple actions are to a standard Intentional mind. However, there is something more to this point than when we said that action always includes a prediction of how the world will evolve spontaneously and as a result of action. The latter capability, however sophisticated, only requires a *forward* projection of the situation currently at sight (and, as far as memories are concerned, meshing this with a *backward* projection). Planning requires instead wholly alternative situations to be conjured up—it is, in the same metaphor, a *lateral* projection.

The example of long-term planning is particularly telling. When we plan where to spend our holidays next year, we use our knowledge of the present: we may assume, for example, that we will be able to count on a certain income, that we will still have the friends we have today, and so on. This is more the identification of certain pillars with which to build the alternate situations than a real prediction of how our life will evolve over the next several months. It is only after building such alternate situations that we begin planning, that is, imagining how *those* situations might evolve.

Thus, the current situation may contain, as if it were transparent, its future development; but it does not contain alternate situations, which have instead to be conjured up from scratch by projecting possible interferences with actual or potential dynamics. Such alternate situations will be dynamic in their turn, which makes the whole operation comparatively difficult. Planning thus requires more than just “basic” Intentional capabilities.

Planning is not something that occurs every now and then, or once in a while. Once a species has such capability, it will play a role in all of its mental activities. So, we are *always* making plans. Everything we do is only understandable as part of a plan: it is because we always live in several situations, only one of which is the “real” one, that we can decide, for example, to sit at a desk writing a paper on a sunny Sunday afternoon. The writer's current situation includes being a professional researcher, pursuing certain intellectual interests, having to earn salary so to be able to keep a certain way of life, and so on; therefore, writing a scientific article is just part of the affordances that she views in the situation. It would make no sense to keep these knowledge, desires, attitudes, and so on, separate from the writer's representation of the situation, or to conceive of them as add-on, “meta-layer” features plugged into an otherwise simpler system. They are just part of the writer's current flows of meaning: to be meta-Intentional requires no additional effort to a meta-Intentional animal.

It follows from this description that a meta-Intentional agent can also try and modify what we have called pillars and see what would happen, like a writer would. Indeed, this is the starting point for fiction, pretend play, story-telling, and the ability to put oneself in somebody else's shoes.

Finally, meta-Intentional descriptions allow an agent to imagine how she would look from the outside. Our control system is, at least within certain limits, an observer of its own interactions. One of the result of such observation is a narrative description of how we would appear from an external standpoint. This capability plays an immediate role in our agency. Many features and dynamics of our social life depend on the internalization of such external descriptions: think for example, of our capability of obeying abstract social rules, of experiencing shame or remorse, of thinking we are overweight, or wondering whether we are sexually attractive.

Furthermore, since our control system uses these observations from the exterior as a feedback on the interaction that is going on under its supervision, with the same spiral dynamics we described above, it can be said to produce, in a sense, behavior, that is a third-person description of our activity. This, again, would not be a separate faculty, but an immediate feature of our Intentionality. Thus, if behavior is conceived of as a function of the observer, and not of the observed organism, then the only animals that really do *behave* are, paradoxically, humans. Which, of course, is what our commonsense knowledge has always taken for granted.

## Conclusion

We are aware that this paper is structured in an unusual way, so let us try again to make our intentions clear. There is a general (albeit, of course, not unanimous) consensus in the cognitive sciences on the very nature of cognition and action. This consensus relies on the substantial irrelevance of consciousness and experience, based on the adoption of either the computational postulate or of various forms of eliminativism. We include neural reductionism in the latter.

Then there are islands and whole archipelagos of dissenting, heterodoxical, and truly heretical positions (for large-scale reviews see e.g., Osbeck, 2009; Manera and Tirassa, 2010; Tirassa and Vallana, 2010). However, while there are reasons to reject what we have called the consensus, the alternatives (still?) show a low tendency to merge into a unitary paradigm, or anyway to give rise to one.

This has been going on for several years now. It is not necessarily worrying: after all, most disputes in philosophy, in psychology, in economics, or in the social sciences appear to be as old as human culture is, and this does not detract from their interest or usefulness. In psychology, which is the most important of our cultural and scientific matrices, eliminativists cohabit more or less happily with phenomenologists, behaviorists with psychoanalysts and computational neuroscientists, and so on.

The downside of this situation, however, is a sense that no dispute can ever be settled, that the same arguments are reiterated over and over like ready-made tokens or gambling chips. What we tried to do with this paper is to look for a way out of this seeming stalemate by avoiding, as much as possible, the umpteenth discussion about the premises and instead trying to just develop the consequences of a certain set of premises. Instead of getting stuck into well-rehearsed arguments, we brought together a variety of literature, from psychology to biology, from philosophy to artificial intelligence and tried to see what would happen if we took certain ideas seriously and tried to develop some of their consequences.

Of course the attempt is only partially successful, to be optimistic; the limits and possible objections of this work are obvious, ranging from the heterogeneity of the sources used (and sometimes the ambiguity of our interpretations thereof) to the difficulty of imagining the empirical counterparts and consequences of our perspective. We might have chosen one single issue, as circumscribed as possible, and tried to develop it in relative isolation, but this would have brought us back to the starting point. Furthermore, we believe that the real interesting topics—the nature of the mind, the nature and relations of perception and action, and so on—cannot be decomposed without losing too much of their significance and import.

We had to start somewhere, and we definitely are not certain to have reached anywhere. Yet, we hope that the reader has found the attempt decently interesting and fruitful.

## Author contributions

All authors listed, have made substantial, direct and intellectual contribution to the work, and approved it for publication.

### Conflict of interest statement

The authors declare that the research was conducted in the absence of any commercial or financial relationships that could be construed as a potential conflict of interest.

## Footnotes

^1^Conventionally (e.g., Searle, 1983), Intentionality as *aboutness* is written with a capital initial, so to immediately distinguish it from the acceptation of *having an intention* or *doing something intentionally*.

^2^It could be objected that “predator” is a word, and that gazelles have no language. Of course we do not think that the gazelle says to itself “Alas, another goddamn lion…alright: time to run.” We have already argued against the identification of semantics and language. It could also be objected that the lion will actually eat the gazelle if it catches it, and that there is nothing semantic in this, and therefore that the lion is objectively a predator. Again, true, but we are concerned here with the gazelle's mind, not with ethology as construed by human scientists: it is unlikely that the gazelle views the ongoing situation in terms of the nutritional habits of Felis leo as they are portrayed on educational TV channels.

^3^Our definition is akin to Pollock's (1993); however, his was cast within a computational perspective instead of a first-person or consciousness-based one, and thus developed in wholly different directions. Analogously, our use of the word situation has nothing to do with situation semantics (Barwise and Perry, 1983). Also, we are aware that a clinical psychologist, a psychoterapist, or a sociologist might object that the locution “…to her liking” is extremely ambiguous under most circumstances.

^4^Of course, such a one-dimensional conception of emotions and motivations is definitely too rough; however, it may do for our current purposes.

^5^In any case, there seem to be no other possibilities. Computational psychology, cast in terms of libraries of operators (that is, of an abstract, objective and predefined ontology of actions), must at a certain point deliver the responsibility for their realization to non-representational capabilities “of a robotic kind” (see for example McDermott, 1987; Harnad, 1994). At the other end of the spectrum, Searle (1983; 1992) claims that a “nonrepresentational Background” is in charge of the task, but then his description thereof remains somewhat mysterious. One of the advantages of the position that we are advocating here is that it makes sense of the relations between phenomenal dynamics, body dynamics, and external dynamics.

^6^We are not claiming that Gibson would agree with our proposals, but only that we have been influenced by our understanding of his work.
